# Management of malignant struma ovarii: is aggressive therapy justified? Case report and literature review

**DOI:** 10.1186/s13044-022-00132-6

**Published:** 2022-08-11

**Authors:** Letiția Leuștean, Maria-Christina Ungureanu, Cristina Preda, Stefana Catalina Bilha, Florin Obrocea, Radu Dănilă, Laura Stătescu, Delia Gabriela Apostol Ciobanu

**Affiliations:** 1 Department of Endocrinology, “Grigore T. Popa” University of Medicine and Pharmacy Iasi, Iasi, Romania; 2 Green Onco-Medical Bucuresti, București, Romania; 3 Department of Surgery, “Grigore T. Popa” University of Medicine and Pharmacy Iasi, Iasi, Romania; 4 Department of Dermatology, “Grigore T. Popa” University of Medicine and Pharmacy Iasi, Iasi, Romania; 5 Department of Pathology, “Grigore T. Popa” University of Medicine and Pharmacy Iasi, Iasi, Romania

**Keywords:** Malignant struma ovarii, Teratoma, Thyroid carcinoma, Surgery, Radioactive iodine ablation

## Abstract

**Background:**

Struma ovarii (SO) is a rare ovarian teratoma containing predominantly thyroid tissue. In rare situations SO may develop malignancy. Most cases of malignant struma ovarii (MSO) are diagnosed after surgical removal, based on histopathological examination. There are still controversies regarding the extent of surgery and postoperative management in MSO, due to its unpredictable behavior, possible risk of metastasis and relatively high rate of recurrence.

**Case Presentation:**

We present the case of a patient diagnosed with a right ovarian cyst discovered incidentally during routine ultrasound examination. Its rapid growth and pelvic MRI raised the suspicion of a neoplastic process. She underwent total hysterectomy and bilateral adnexectomy. The anatomopathological diagnosis was MSO with follicular variant of papillary thyroid carcinoma. Prophylactic total thyroidectomy was performed, followed by radioactive iodine ablation (RAI), and suppressive therapy with levothyroxine. At 1 year follow-up, the patient was disease free.

**Conclusions:**

Even if latest literature reports consider that completion of local surgery with total thyroidectomy and RAI might be too aggressive in cases of MSO without extraovarian extension, in our case it was decided to follow the protocol for primary thyroid carcinoma, in order to reduce the recurrence risk.

## Background

Struma ovarii (SO) is a rare monodermal variant of ovarian teratoma that contains predominantly thyroid tissue (> 50%), accounting for 0.5–1% of all ovarian tumors and 2–5% of ovarian teratomas [1–4]. It may present any form of thyroid pathology, and in most cases is a benign condition, but approximately 5–10% of these tumors become malignant (MSO), with papillary and follicular carcinoma most common [5, 6].

MSO may be asymptomatic, usually discovered incidentally during routine ultrasound examination, and is difficult to recognize based on clinical or imaging criteria. Most cases are diagnosed after surgical removal of an ovarian cyst or mass, on histopathological examination [6–9].

When symptoms are present, they are non-specific, and may mimic an ovarian cancer. In 5–8% of cases, SO may present with symptoms of hyperthyroidism and goiter [7–10].

The management of MSO is surgical removal, but there are still controverses regarding the extension of surgery. Aggressive treatment with local conservative surgery, followed by total thyroidectomy, radioactive iodine (RAI) and thyroid-stimulating hormone (TSH) suppressive therapy with levothyroxine is an option advocated by many authors, but controversial [1, 11, 12]. Other authors consider that MSO, similar to primary thyroid carcinoma, is a mild type of cancer with low risk of mortality [11].

Being a rare tumor with no consensus regarding its management, some authors proposed different risk stratification that could help to choose the appropriate therapy, including tumor dimensions, histopathological characteristics, or molecular profiling [1, 11].

Although recent studies have shown that MSO has a very good survival outcome, its behavior remains unpredictable, with a relatively high rate of recurrence that does not correlate with the morphology. This makes the decision of therapy sometimes difficult [1, 11].

We report the case of a patient with morbid obesity and incidentally discovered MSO during the preoperative workup for gastric sleeve; the latest highlights regarding the clinicopathologic features, diagnostic criteria, management, and follow-up of this rare tumor are also reviewed.

## Case presentation

**Fig. 1:**
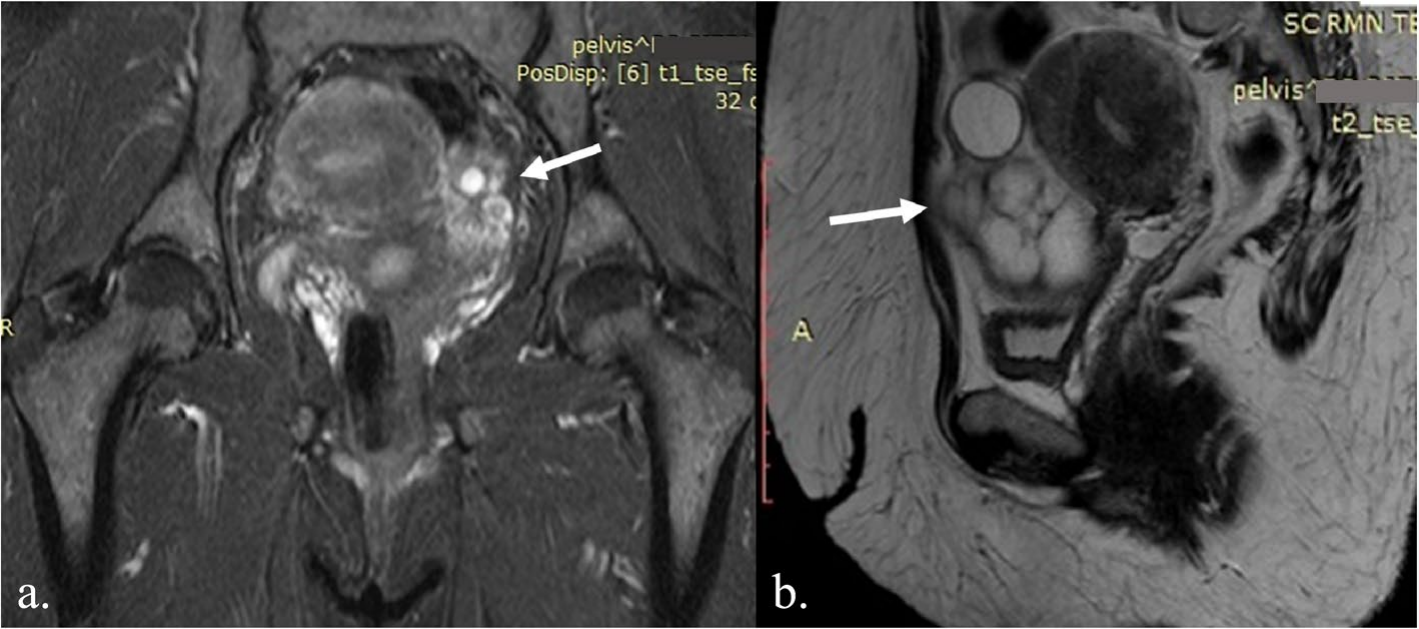
Pelvic MRI showing right adnexal mass with median development, mixed predominantly cystic structure, multiloculated, multiseptated, with reduced solid component, with dimensions of 79 × 66x72 mm (white arrow); **a**. Coronal MRI: T1-weighted image showing variable signal intensity for the cystic component (high and intermediate), and intermediate signal intensity for the solid component of SO; **b**. Sagittal MRI: T2-weighted image showing high signal intensity for the cystic component and low signal intensity for the solid component (septations and walls)

We present the case of a 46-year-old multiparous (parity 2) patient, with normal menstrual cycle and a personal history of morbid obesity, admitted in a specialized service to perform the preoperative assessment for gastric sleeve. Pelvic ultrasound revealed a large right ovary (57 × 38 mm) with a cystic image relatively well defined in the periphery, with dimensions of approximately 44 × 43 mm, and a normal appearance of the left ovary. Six months after bariatric surgery, abdominopelvic ultrasound showed an increase in the size of the right ovarian cyst, and a pelvic magnetic resonance imaging (MRI) was performed, revealing a right adnexal mass with median development, mixed predominantly cystic structure, multiloculated, multiseptated, with reduced solid component, with dimensions of 79 × 66x72 mm. The tumor showed development up to the level of the anterior abdominal wall, with no limit of separation with the visceral peritoneum over a length of approximate 5 mm. In addition, two nodules with intense signal (the largest of about 5.5 mm), and other small lymph node images (maximal diameter of 6 mm) posterior to the external iliac vascular bundle were found (Fig. 1).

The physical examination didn’t reveal any palpable pelvic mass, and the patient didn’t complain of abdominal or pelvic pain. The patient had a personal medical history of right ovarian cyst resected ten years before (benign histopathological examination), arterial hypertension, no history of thyroid disease, and a family history of neoplasia (mother with cervical neoplasm and father with pulmonary neoplasm).

In the diagnostic workup, the serum tumor marker cancer antigen-125 (CA-125) was performed, with slightly elevated levels at 80 UI/mL (reference values < 35 UI/mL), thus raising the suspicion of ovarian neoplasm. Preoperative thyroid function tests revealed a TSH of 1.21 µUI/mL (reference interval: 0.4–4 µUI/mL), and a free thyroxine (fT4) of 0.94 ng/dL (reference interval: 0.89–1.76 ng/dL).

Exploratory laparotomy found a right adnexal mass of approximate 120 mm, well-defined, without tendency to local invasion. The uterus and left ovary had normal macroscopic appearance, as well as the rest of the peritoneal cavity. Subsequently, a right adnexectomy was performed and the intra-operative frozen section consult revealed a benign ovarian tumor-SO. The surgery was completed with total hysterectomy and left laparoscopic adnexectomy. Her postoperative evolution was uneventful, and she was discharged after two days.

**Fig. 2:**
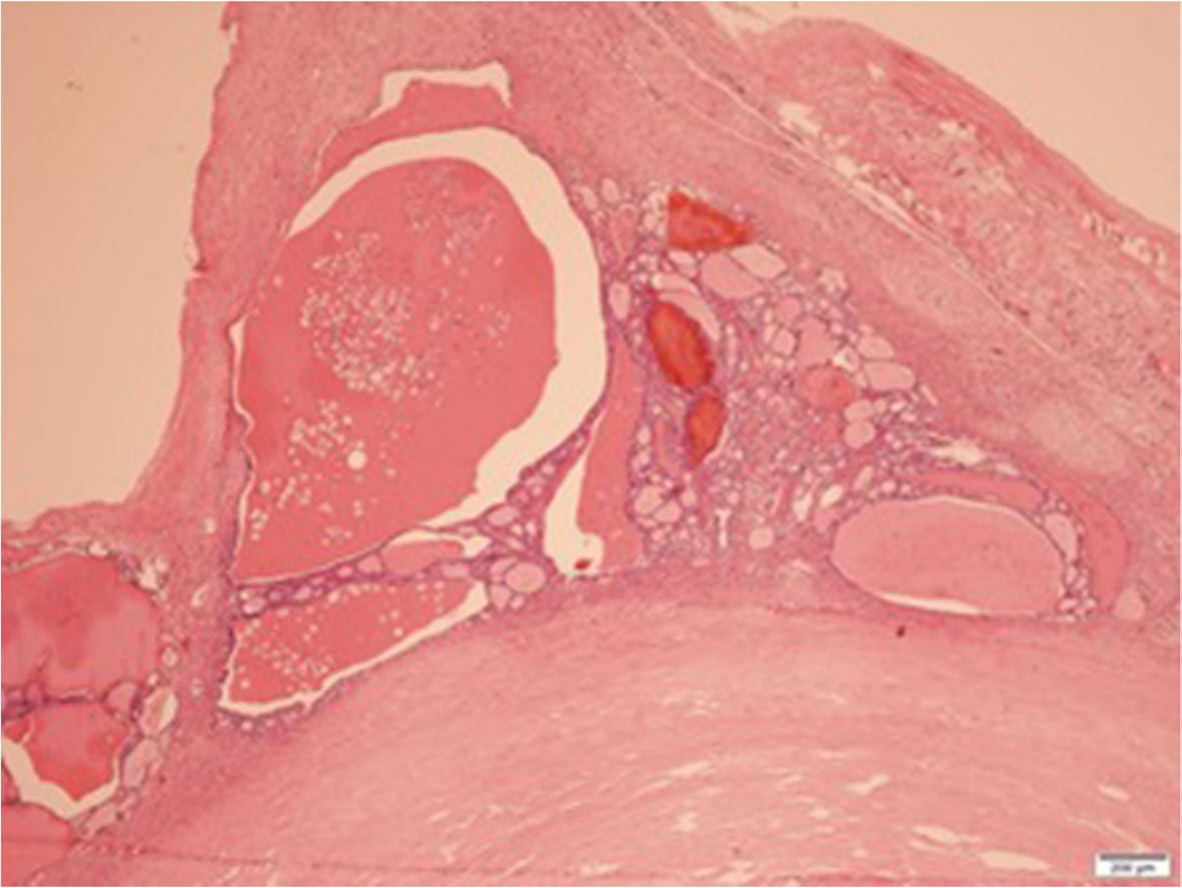
Follicles with variable dimensions, covered by one layer of cells, filled with colloid (HE, × 40). HE = Haematoxylin and eosin staining

**Fig. 3:**
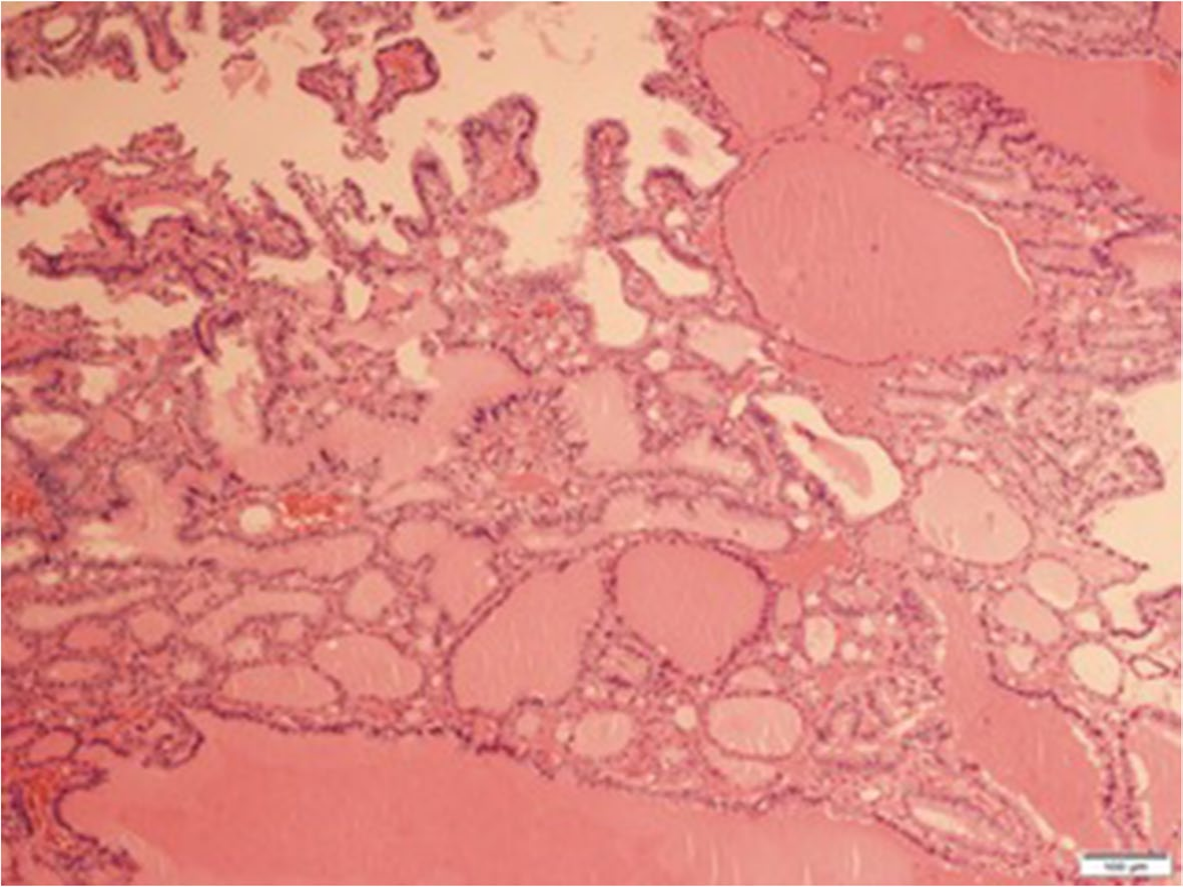
Area with malignant changes (center of the image), with predominantly follicular pattern, isolated papillae and clear, vesicular nuclei specific for papillary malignant tumor (HE, × 100). HE = Haematoxylin and eosin staining

**Fig. 4:**
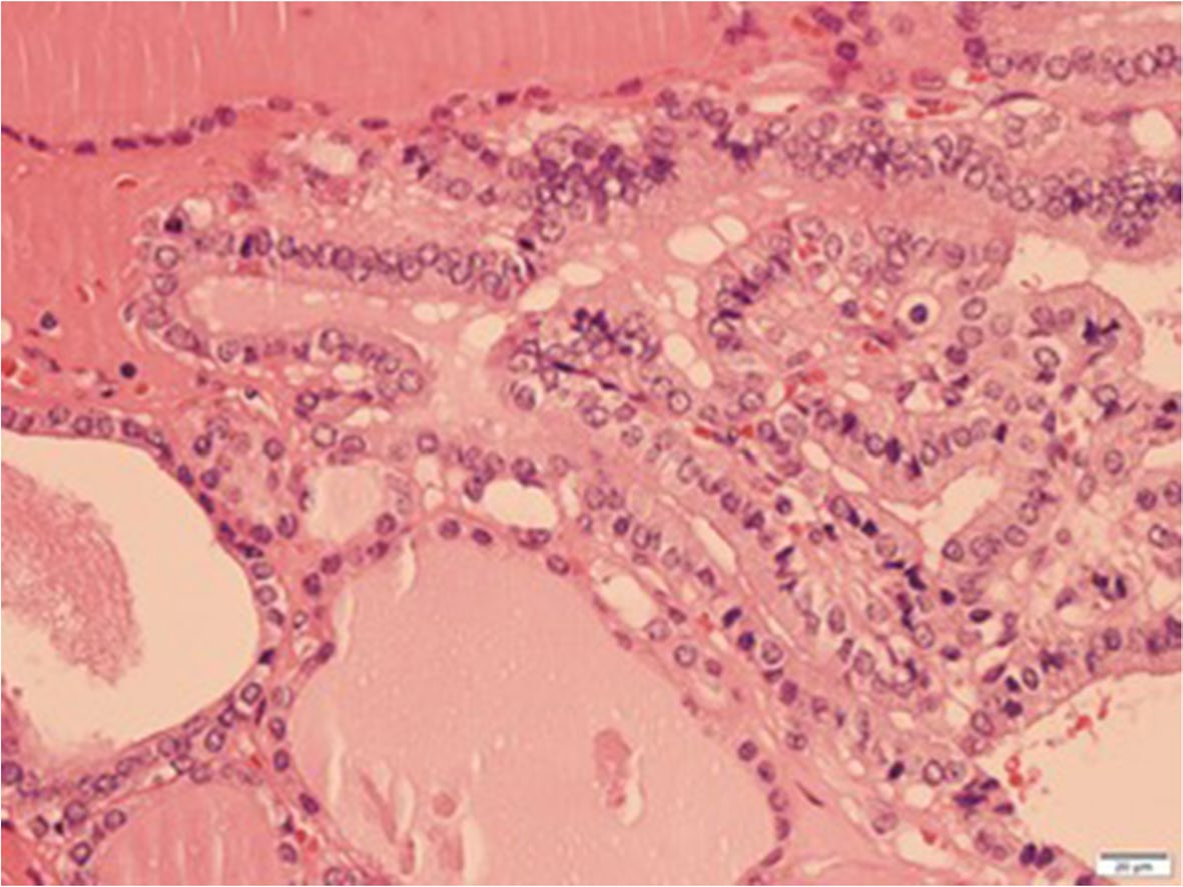
Clear, vesicular nuclei specific for papillary malignant tumor and isolated mitoses (HE, × 400). HE = Haematoxylin and eosin staining

**Fig. 5:**
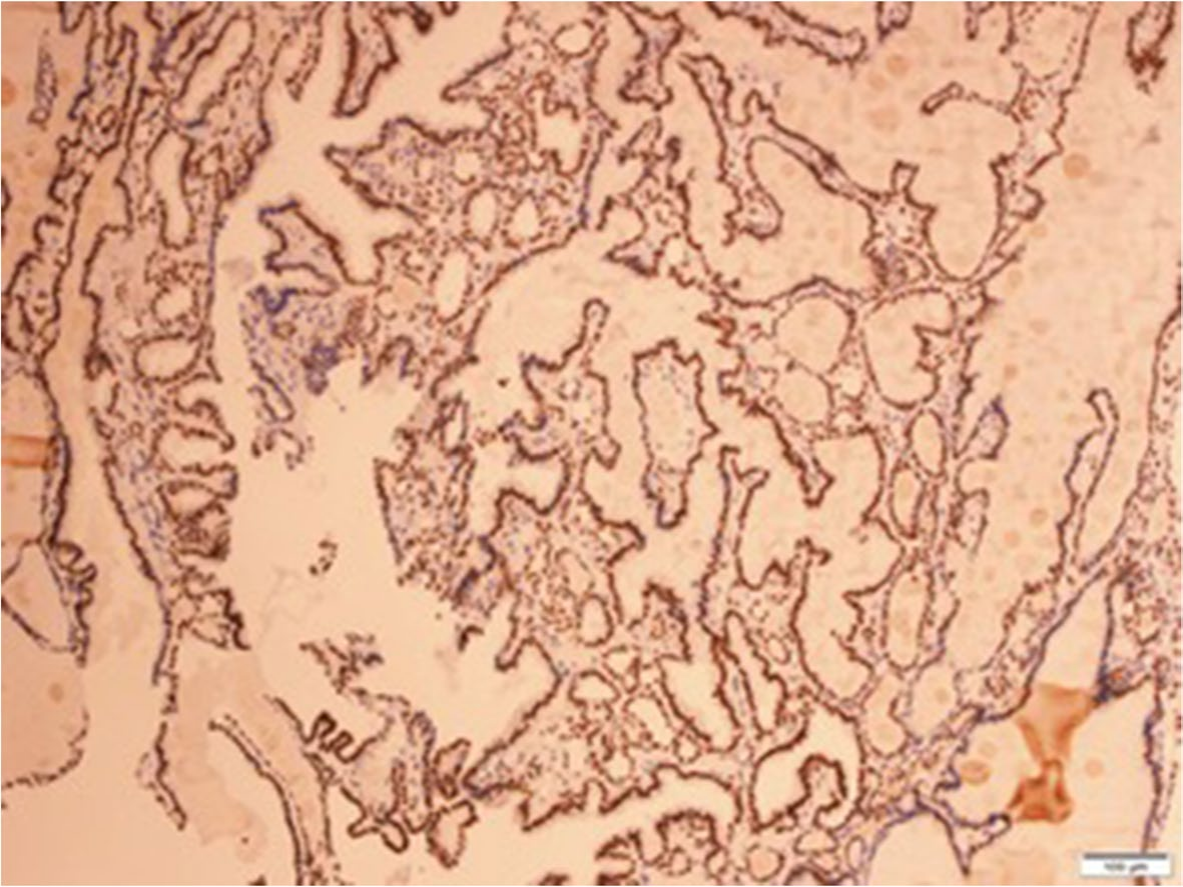
Diffuse, strong nuclear positivity at immunohistochemical staining for TTF1, consistent with the diagnosis of ovarian thyroid tissue (TTF1, × 10). HE = Haematoxylin and eosin staining

Gross examination revealed a 15 mm size fibroid nodule in the body of the uterus and a right ovarian cyst of 90 mm with smooth capsule. Cut section of the right ovarian cyst showed multilocular cystic area filled with turbid, yellow liquid, solid walls with thickness varying between 1–4 mm, and no intracystic papillary projections. Histopathological examination revealed (1) an interstitial leiomyoma of the uterus; (2) a serous ovarian cystadenofibroma; (3) a monodermal ovarian teratoma with thyroid tissue characteristic of SO, associated with a limited area of 7 mm (maximum diameter) with distorted follicles, with the formation in some places of papillary structures, displaying enlarged, optically clear nuclei, nuclear grooves, overlapping nuclei and also similar microscopic foci distantly located from the described proliferation – all consistent with the diagnosis of follicular variant of thyroid papillary carcinoma (Fig. 2, 3, 4). Immunohistochemistry in tumoral cells showed positivity for the specific marker thyroid transcription factor (TTF-1), consistent with the diagnosis of ovarian thyroid tissue (Fig. 5).

Postoperative thyroid function tests, thyroid-specific autoantibodies (anti-thyroid peroxidase and anti-thyroglobulin antibodies) and calcitonin levels were all within normal limits. Additional cervical ultrasound was performed, revealing a thyroid gland with normal structure and echogenicity.

**Fig. 6:**
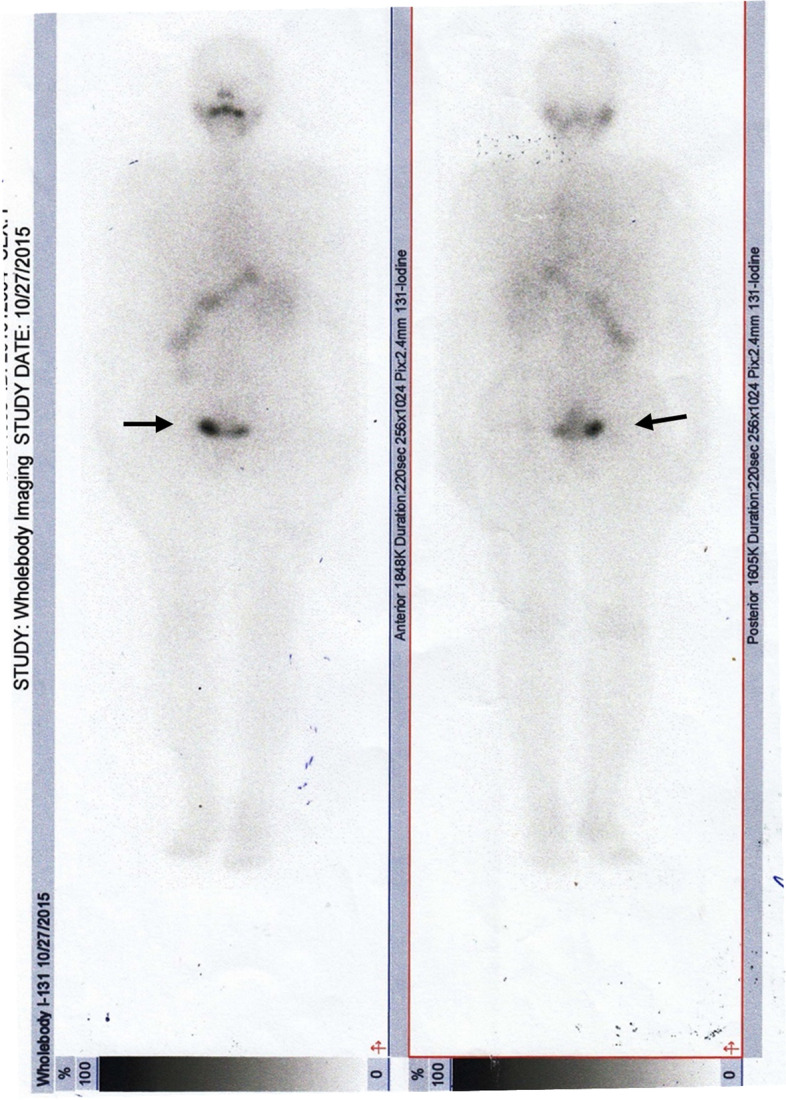
Post-therapeutic I-131 whole body scan – anterior view (left) and posterior view (right). Remnant uptake in the pelvic area after iodine radiopharmaceutical washout (black arrows)

Considering the pathological peculiarity of the teratoma and the lack of a well-defined consensus for the management of this pathology at that time, it was decided in a multidisciplinary team to follow the therapeutic protocol for primary thyroid carcinoma. Consequently, prophylactic total thyroidectomy was performed, followed by RAI (90 mCi I-131). The histopathological examination revealed an adenomatous colloidal goiter with embryonic remnants. Postoperative stimulated thyroglobulin (Tg) level was 0.65 ng/mL (reference range for athyreotic patients: < 0.04 ng/mL), with anti-thyroglobulin antibodies (TgAb) level < 10 UI/mL (reference range: < 115 UI/mL), and a TSH of 93.64 µUI/mL. Post therapeutic I-131 whole-body scan (WBS) revealed a small uptake in the area of the right ovary (Fig. 6). The patient started TSH suppressive therapy with levothyroxine 150 µg/day. The six months follow-up confirmed good oncologic control, with undetectable Tg levels (serum Tg < 0.04 ng/mL, TSH = 58.88 µUI/mL) and the pelvic MRI didn’t reveal any pathological sign. Tumor recurrence was also excluded at 1 year follow-up, the patient being considered disease-free for primary differentiated thyroid carcinoma.

## Discussion

MSO is a very rare tumor, accounting for approximate 1% of mature teratomas, with a median age at diagnosis between 40 and 50 years, and the highest incidence in premenopausal women aged between 30–40 years [1, 11]. Even though the first case was described more than 120 years ago, less than 200 cases have been reported until now [1, 11, 13–15]. ”Pure” MSO consists entirely of thyroid carcinoma, otherwise it is named ”impure”, with a mixed structure of teratoma components and thyroid carcinoma. ”Impure” MSO is far more frequent than ”pure” MSO [11].

In most cases, MSO is discovered incidentally during routine ultrasonography, due to the lack of specificity regarding clinical or imaging criteria [6, 9, 10]. Retrospective studies have shown that as much as 40% of patients are asymptomatic, the diagnosis being often delayed [11, 16]. When symptoms are present, they are non-specific and may overlap with those of ovarian cancer. Most reported symptoms are lower abdominal pain, palpable abdominal mass, bloating, urinary incontinence, vaginal bleeding, and ascites [6, 7, 9–11]. Ascites has been described in 17–33.3% of patients, generally without malignant cells [9, 11, 17]. The presence of obvious symptoms may be an indicator of distant metastases [11]. In rare cases (5–8%), SO may present with hyperthyroidism and goiter, or Graves’ disease [5, 8, 9]. In a recent research, the percentage of patients with hyperthyroidism and MSO was even lower than previous literature reports (2.78%) [11].

In our case, the patient was asymptomatic, with regular menses, and normal thyroid function, the tumor being discovered incidentally during the routine workup for bariatric surgery. The initial diagnosis was right ovarian cyst, but its rapid growth in the next 6 months required a pelvic MRI that raised the suspicion of a neoplastic process.

The role of imaging in the diagnosis of SO is still limited, but of major importance in order to avoid an aggressive management, such as cancer type surgery with total hysterectomy, bilateral adnexectomy, omentectomy, and even appendectomy [10]. Because it’s a rare tumor with a variety of non-specific appearances, the differential diagnosis with a mature teratoma, cystadenoma, cystadenocarcinoma, endometriosis, or metastasis is often challenging [10, 18].

The classic ultrasound appearance of SO is that of a multilocular ovarian cyst, with variable amounts of well-vascularized solid components on color Doppler [5, 10]. The appearance of SO on MRI may overlap with that of an ovarian epithelial carcinoma, presenting as a mixed tumor, with multi-cystic and solid components with variable signal intensity, or as an adnexal mass, accompanied by ascites [10, 19]. Unlike cystic areas, which can present both high and low signal intensity on T1 and T2-weighted images, the thyroid tissue within SO—which represents the solid component—has intermediate signal intensity on T1, and low signal intensity on T2-weighted images [10, 20, 21].

The variability of the signal intensity in the cystic areas on MRI is considered by most authors a characteristic of SO; it is a consequence of the presence of viscous colloid in the follicles of struma, due to the condensation of thyroglobulin and thyroid hormones within the colloid [10, 21–24]. The classic described”lacy” pattern on MRI is due to the multilobulate surface of the mixed tumor, with solid and cystic components with low signal intensity on T2 and intermediate signal on T1-weighted images, but with strong enhancement of the solid components, including thickened walls and septations [10, 20, 21]. Unfortunately, there are no specific characteristics for detecting MSO on MRI, except for the rapid growth of an invasive and inhomogeneous large tumor, with irregular borders [10, 25]. The presence of ascites is not an indicator of malignancy, as it may be also present in benign SO [10].

The rapid evolution of the tumor, the large dimensions, and the apparent invasive character on the visceral peritoneum raised the suspicion of an ovarian neoplasia in our patient. In this context, CA-125, a classical tumor marker for ovarian cancer was performed, and a slightly elevated value of 80 UI/mL was found (reference values < 35 UI/mL), thus supporting the suspicion of malignancy.

Even though CA-125 is increased in 80% of epithelial ovarian carcinomas, its specificity in the diagnosis is limited, as this marker may be elevated also in other malignancies (endometrium, breasts, lungs), as well as in endometriosis, or even in physiologic states like menstruation or pregnancy [9, 26]. Reports have shown that CA-125 is not a specific tumor marker for MSO, an important percentage of cases having normal levels of CA-125 [9–11, 26]. In a recent research performed on 144 published cases of MSO, only 51.6% had high levels of CA-125 [11]. Elevated levels of CA-125 may also be present in SO, a possible explanation being the presence of ascites with consecutive inflammation of peritoneum and pleura [9, 10, 26].

Most cases of SO are diagnosed after surgical removal of an ovarian cyst or mass, based on histopathological examination [6–9]. The thyroid tissue within SO is morphologically similar to that of cervical thyroid gland, and thus may present any thyroid pathology, including malignancy [4, 5].

Literature reports indicate that the most frequent type of carcinoma found in MSO is papillary thyroid carcinoma (PTC), accounting for approximate 40–50% of cases, followed by follicular thyroid carcinoma, follicular variant of papillary thyroid carcinoma (FVPTC), mixed follicular-papillary thyroid carcinoma, and in very rare cases anaplastic and medullary carcinoma [1, 11]. Most authors agree that the same histopathological criteria as for thyroid carcinoma must be used for the diagnosis of MSO. Thus, for MSO with PTC, the histopathologic diagnosis is based on the presence of typical features such as”ground glass”, overlapping nuclei, nuclear grooves, and sometimes papillary architecture [4, 5, 11]. Similar to primary follicular thyroid carcinoma, the recognition of MSO with follicular thyroid carcinoma is more difficult, and the criteria used are capsular and vascular invasion, infiltration in the surrounding organs, and distant metastasis [4, 5, 11]. Given the fact that the ovarian tumor does not usually have a well-defined capsule, the infiltration of the ovarian tissue, along with other features described before, are used as criteria for malignancy [4, 5, 11]. The diagnosis of MSO with FVPTC is based on the presence of nuclear features characteristic for PTC, in the absence of papillary architecture [5, 11]. For the less differentiated forms of thyroid carcinoma in MSO, the histopathological criteria used for diagnosis are the presence of nuclear atypia, structural and architectural distortions, and high mitotic activity [5].

It is very important to differentiate MSO from other ovarian tumors that may have some similarities on histopathological examination, such as cystadenocarcinoma, Brenner tumor, or granulosa tumor. Performing thyroid tissue specific immunohistochemical markers, like thyroglobulin or TTF-1, allows the differential diagnosis [5]. In doubtful cases, immunohistochemical markers help distinguish between benign and malignant thyroid tissue in SO: some authors have proposed cytokeratin 19 (CK 19), galectin-3, HBME-1, and CD 56, as valuable markers with a 100% specificity for MSO [5, 11, 27, 28].

An important issue in the diagnosis and management of MSO is also the exclusion of a metastasis from primary thyroid cancer to the ovary. The radiologic finding of a mixed tumor with cystic and well-vascularized solid components on color Doppler ultrasonography, and high signal enhancement on MRI examination, may help in the differential diagnosis [5, 11, 29].

Clinical examination and ultrasonography of the thyroid gland is also mandatory to exclude a multifocal primary thyroid carcinoma [5, 11, 30]. In a recent metanalysis which included 144 patients with MSO, only 8 (5.56%) had primary PTC, and most authors considered that the two forms of malignancy were histologically different, while the eventuality of an ovarian metastasis from a primary thyroid carcinoma is very rare [5, 11, 30]. Although there is no consensus on the management of MSO, the coexistence of a suspicious lesion in the cervical thyroid tissue requires aggressive treatment with total thyroidectomy, and even RAI [11, 30].

In our case, the exploratory laparotomy with right adnexectomy was followed by laparoscopic total hysterectomy and left adnexectomy, due to the suspicion of ovarian malignancy. The diagnosis of”impure” MSO with FVPTC was confirmed by the presence of characteristic histopathological features and positive immunohistochemistry for TTF-1 in tumoral cells. Thyroid gland pathology was excluded by ultrasonography and thyroid function tests, which were within normal limits. There were no signs of local or distant metastasis. Based on literature data at that moment and the lack of consensus regarding the management of MSO, the therapeutic protocol for primary thyroid carcinoma was applied. Consequently, prophylactic total thyroidectomy, followed by RAI were performed.

Even though the standard management of MSO is surgical resection, there are still controversies regarding the extent of surgery, postoperative treatment, and follow-up of this rare pathology [11, 31]. Total abdominal hysterectomy and bilateral salpingo-oophorectomy in postmenopausal women, and conservative surgery with unilateral oophorectomy or even limited tumor resection for young women who desire fertility are the main choices in most studies. Adjuvant therapy with total thyroidectomy, followed by RAI and suppressive therapy is an option advocated by several authors, in order to prevent recurrence, in cases of advanced disease with metastasis, or when fertility preservation is not required [11, 12, 31].

Literature reports metastasis of SO in approximate 5–23% of cases, even after 26 years from the diagnosis: the most frequently encountered are intra-abdominal, but are also found in the lungs, bones, and central nervous system [1, 11, 30, 31]. The reported recurrence rate is also variable, ranging from 7.5% to 22–35% in different studies [1].

Due to the rarity of this tumor and the lack of a consensus regarding its management and follow-up, some authors proposed different risk stratification that could help to choose the appropriate therapy. While Yassa et al. [32] proposed that tumors larger than 2 cm, with extra-ovarian extension and aggressive histopathological characteristics must be considered at high risk, needing completion of surgery with total thyroidectomy and RIA, Janszen et al. [33] suggested that even smaller MSO (< 1 cm) should benefit from this aggressive therapy [11, 32, 33].

It is well known that most Tg is secreted by the thyroid gland. Prophylactic thyroidectomy to exclude a primary thyroid carcinoma followed by RAI will allow for Tg to be a reliable marker for metastasis or relapse [1, 11]. However, later studies raised questions about the necessity of RAI after pelvic surgery in patients with MSO confined to the ovary, without metastasis [1, 34, 35]. In a recent meta-analysis, even though the overall recurrence rate in patients with MSO treated with RAI was significantly lower than in patients without RAI, the overall survival rate was not improved; the authors concluded that this aggressive therapy is not mandatory for MSO confined to the ovary [1]. Moreover, another recent research demonstrated that radioiodine therapy in MSO didn’t improve the mortality rate, questioning again its necessity in this form of MSO [11]. Although the recurrence rate in MSO confined to the ovary remains relatively high, with the most recently reported being as high as 21.8% after a median of 14 years, the overall survival rate is excellent. Authors concluded that MSO, similar to cervical thyroid carcinoma, is a mild type of cancer with low risk of mortality [1, 11]. The same research concluded that the outcome of MSO may be related to the type of carcinoma, age of the patient, and the presence of metastasis, all these possible risk factors helping to choose the right therapeutical approach [11].

Knowing that molecular profiling may help to assess risk stratification in cervical thyroid carcinoma, researchers have tried to identify different somatic mutations that could predict clinical outcome in MSO. Until now, there are limited published data on MSO harboring BRAF and RAS mutations and RET/PTC rearrangements. Coexistence of these mutations with other genomic anomalies such as telomerase reverse transcriptase (TERT) promoter mutations, may have a synergistic effect on increasing the risk of recurrence in MSO, but further studies are needed to evaluate the impact of molecular profile in making the right therapeutic decision [1, 36, 37].

Data from recent reports indicate that the mainstays in the follow-up of patients with MSO should be the concomitant assessment of serum Tg and TgAb, along with thyroid and pelvic imaging, even in the absence of thyroidectomy. Any raise in serum Tg or TgAb values in patients with thyroidectomy and RAI, or elevated serum Tg values above baseline in patients with preserved thyroid indicate the need for further evaluation for recurrence [1, 38].

Because the behavior of MSO might be unpredictable, with an elevated recurrence risk after many years of evolution, prophylactic total thyroidectomy, and RAI, followed by iodine scanning and Tg assessment for relapse detection were considered the best approach in our patient at that time. The histopathological examination of the thyroid gland didn’t reveal any form of malignancy, and postoperatively value of stimulated Tg was less than 1 ng/mL, but post therapeutic I-131 WBS showed a small uptake in the area of the left ovary, reinforcing the belief that aggressive therapy was the optimal choice for this patient. Her outcome was excellent, with undetectable disease at 6 months and 1 year follow-up.

## Conclusions

MSO is a rare ovarian teratoma with unpredictable behavior, possible risk of metastasis and relatively high rate of recurrence. The preoperatively diagnosis remains difficult, most cases being recognized on histopathological examination. The differential diagnosis with other forms of ovarian neoplasia is very important, in terms of management and evolution. Being a very rare tumor, there is still no consensus regarding the optimal management and follow-up. Latest literature reports consider that completion of local surgery with total thyroidectomy and RAI might be too aggressive in cases of MSO without extraovarian extension; however, in our case, the protocol for primary thyroid carcinoma was applied in order to reduce the recurrence risk and to allow monitoring via Tg levels. The patient was disease-free at 1 year follow-up.

## Data Availability

Data sharing is not applicable to this article as no datasets were generated or analyzed during the current study.

## References

[1] Li S, Yang T, Xiang Y, Li X, Zhang L, Deng S (2021). Clinical characteristics and survival outcomes of malignant struma ovarii confined to the ovary. BMC Cancer.

[2] Fujiwara S, Tsuyoshi H, Nishimura T, Takahashi N, Yoshida Y (2018). Precise preoperative diagnosis of struma ovarii with pseudo-Meigs’ syndrome mimicking ovarian cancer with the combination of 131 I scintigraphy and 18 F-FDG PET: case report and review of the literature. J Ovarian Res.

[3] Aguilera BG, Vazquez RG, Herguido NG, Gallego FS, Gonzalez EN (2015). The lack of consensus in management of malignant struma ovarii. Gynecol Endocrinol.

[4] Selvaggi F, Risio D, Waku M, Simo D, Angelucci D, D’Aulerio A (2012). Struma ovarii with follicular thyroid-type carcinoma and neuroendocrine component: case report. World J Surg Oncol.

[5] Salman WD, Singh M, Twaij Z (2010). A case of papillary thyroid carcinoma in struma ovarii and review of the literature. Patholog Res Int.

[6] Rockson O, Kora C, Ramdani A, Basma A, Bouhout T, Serji B (2020). Struma ovarii: two case reports of a rare teratoma of the ovary. J Surg case reports..

[7] Al Hassan MS, Saafan T, El Ansari W, Al Ansari AA, Zirie MA, Farghaly H, et al. The largest reported papillary thyroid carcinoma arising in struma ovarii and metastasis to opposite ovary: case report and review of literature. Thyroid Res. 2018;11(1). 10.1186/S13044-018-0054-9 10.1186/s13044-018-0054-9 PMC6056926 30061934

[8] Koehler VF, Keller P, Waldmann E, Schwenk N, Kitzberger C, Schmohl KA, et al. An unusual case of struma ovarii. Endocrinol diabetes Metab case reports. 2021;2021(1). 10.1530/EDM-20-0142 10.1530/EDM-20-0142 PMC7983514 33682680

[9] Yoo S-C, Chang K-H, Lyu M-O, Chang S-J, Ryu H-S, Kim H-S (2008). Clinical characteristics of struma ovarii. J Gynecol Oncol.

[10] Dujardin MI, Sekhri P, Turnbull LW (2014). Struma ovarii: role of imaging?. Insights Imaging.

[11] Cui Y, Yao J, Wang S, Zhao J, Dong J, Liao L (2021). The Clinical and Pathological Characteristics of Malignant Struma Ovarii: An Analysis of 144 Published Patients. Front Oncol..

[12] Wee JYS, Li X, Chern BSM, Chua ISY (2015). Struma ovarii: management and follow-up of a rare ovarian tumour. Singapore Med J.

[13] Dardik RB, Dardik M, Westra W, Montz FJ (1999). Malignant struma ovarii: two case reports and a review of the literature. Gynecol Oncol.

[14] Goffredo P, Sawka AM, Pura J, Adam MA, Roman SA, Sosa JA (2015). Malignant struma ovarii: a population-level analysis of a large series of 68 patients. Thyroid.

[15] Siegel MR, Wolsky RJ, Alvarez EA, Mengesha BM (2019). Struma ovarii with atypical features and synchronous primary thyroid cancer: a case report and review of the literature. Arch Gynecol Obstet.

[16] Wolff EF, Hughes M, Merino MJ, Reynolds JC, Davis JL, Cochran CS (2010). Expression of benign and malignant thyroid tissue in ovarian teratomas and the importance of multimodal management as illustrated by a BRAF-positive follicular variant of papillary thyroid cancer. Thyroid.

[17] Robboy SJ, Shaco-Levy R, Peng RY, Snyder MJ, Donahue J, Bentley RC (2009). Malignant struma ovarii: An analysis of 88 cases, including 27 with extraovarian spread. Int J Gynecol Pathol.

[18] Shanbhogue AKP, Shanbhogue DKP, Prasad SR, Surabhi VR, Fasih N, Menias CO (2010). Clinical syndromes associated with ovarian neoplasms: a comprehensive review. Radiographics.

[19] Park SB, Kim JK, Kim KR, Cho KS (2008). Imaging findings of complications and unusual manifestations of ovarian teratomas. Radiographics..

[20] Shen J, Xia X, Lin Y, Zhu W, Yuan J (2011). Diagnosis of Struma ovarii with medical imaging. Abdom Imaging.

[21] Matsuki M, Kaji Y, Matsuo M, Kobashi Y (2000). Struma ovarii: MRI findings. Br J Radiol.

[22] Matsumoto F, Yoshioka H, Hamada T, Ishida O, Noda K (1990). Struma ovarii: CT and MR findings. J Comput Assist Tomogr.

[23] Yamashita Y, Hatanaka Y, Takahashi M, Miyazaki K, Okamura H (1997). Struma ovarii: MR appearances. Abdom Imaging.

[24] Joja I, Asakawa T, Mitsumori A, Nakagawa T, Hiraki Y, Kudo T (1998). Struma ovarii: appearance on MR images. Abdom Imaging.

[25] Loizzi V, Cormio G, Resta L, Fattizzi N, Vicino M, Selvaggi L (2005). Pseudo-Meigs syndrome and elevated CA125 associated with struma ovarii. Gynecol Oncol.

[26] Jin C, Dong R, Bu H, Yuan M, Zhang Y, Kong B (2015). Coexistence of benign struma ovarii, pseudo-Meigs’ syndrome and elevated serum CA 125: Case report and review of the literature. Oncol Lett.

[27] Muthusamy S, Azhar Shah S, Abdullah Suhaimi SN, Kassim N, Mahasin M, Mohd Saleh MF (2018). CD56 expression in benign and malignant thyroid lesions. Malays J Pathol.

[28] Pyo JS, Kim DH, Yang J (2018). Diagnostic value of CD56 immunohistochemistry in thyroid lesions. Int J Biol Markers.

[29] Wang J, Luo H, Yang T, Yang F, Tian T (2017). Imaging features of struma ovarii in conventional ultrasound and CEUS. Chinese J Med Imaging Technol.

[30] Leong A, Roche PJR, Paliouras M, Rochon L, Trifiro M, Tamilia M (2013). Coexistence of malignant struma ovarii and cervical papillary thyroid carcinoma. J Clin Endocrinol Metab.

[31] Kumar SS, Rema P, R AK, Varghese BT (2014). Thyroid type papillary carcinoma arising in a mature teratoma. Indian J Surg Oncol..

[32] Yassa L, Sadow P, Marqusee E (2008). Malignant struma ovarii. Nat Clin Pract Endocrinol Metab.

[33] Janszen EWM, Van Doorn HC, Ewing PC, De Krijger RR, De Wilt JHW, Kam BLR (2008). [Malignant struma ovarii]. Ned Tijdschr Geneeskd.

[34] Marti JL, Clark VE, Harper H, Chhieng DC, Sosa JA, Roman SA (2012). Optimal surgical management of well-differentiated thyroid cancer arising in struma ovarii: a series of 4 patients and a review of 53 reported cases. Thyroid.

[35] McGill JF, Sturgeon C, Angelos P (2009). Metastatic struma ovarii treated with total thyroidectomy and radioiodine ablation. Endocr Pract.

[36] Poli R, Scatolini M, Grosso E, Maletta F, Gallo M, Liscia D (2021). Malignant struma ovarii: next-generation sequencing of six cases revealed Nras, Braf, and Jak3 mutations. Endocrine.

[37] Tan A, Stewart CJR, Garrett KL, Rye M, Cohen PA (2015). Novel BRAF and KRAS Mutations in Papillary Thyroid Carcinoma Arising in Struma Ovarii. Endocr Pathol.

[38] Filetti S, Durante C, Hartl D, Leboulleux S, Locati LD, Newbold K (2019). Thyroid cancer: ESMO Clinical Practice Guidelines for diagnosis, treatment and follow-up†. Ann Oncol Off J Eur Soc Med Oncol.

